# Progressive Response to Repeat Application of Capsaicin 179 mg (8% w/w) Cutaneous Patch in Peripheral Neuropathic Pain: Comprehensive New Analysis and Clinical Implications

**DOI:** 10.1093/pm/pnab113

**Published:** 2021-04-19

**Authors:** Rainer Freynhagen, Charles Argoff, Mariëlle Eerdekens, Sylvia Engelen, Serge Perrot

**Affiliations:** 1 Department of Anaesthesiology, Critical Care Medicine, Pain Therapy and Palliative Care, Benedictus Hospital Feldafing, Feldafing, Germany; 2 Department of Anaesthesiology, Klinikum Rechts der Isar, Technische Universitat Munchen, Munich, Germany; 3 Department of Neurology, Albany Medical College, Comprehensive Pain Center, Albany Medical Center, Albany, New York, USA; 4 Grünenthal GmbH, Aachen, Germany; 5 Université de Paris, Hôpital Cochin, INSERM U987, Paris, France

**Keywords:** Topical Capsaicin, Diabetic Neuropathies, Peripheral Neuropathies

## Abstract

**Objective:**

To investigate the efficacy of repeated application of capsaicin 179 mg cutaneous patch in nonresponders to the first application.

**Design:**

Post hoc, as-treated analysis of two prospective trials (STRIDE and PACE) with 52-week follow-up.

**Blinding:**

Open-label.

**Setting:**

Multicenter clinical trial.

**Subjects:**

STRIDE: nondiabetic neuropathic pain; PACE: painful diabetic peripheral neuropathy.

**Methods:**

Patients were divided according to number of applications needed before attainment of a ≥30% reduction in average pain intensity (question 5 of the Brief Pain Inventory [BPI-Q5]). We assessed the change from baseline in average pain intensity (BPI-Q5), mean “interference with sleep” score, Patient Global Impression of Change, quality of life (QOL) via the EuroQol 5-dimension, and Self-Assessment of Treatment.

**Results:**

In STRIDE and PACE, respectively, n = 306 and n = 313 received the capsaicin patch; n = 60 and n = 96 had a response after the first application, n = 33 and n = 68 after the second, and n = 11 and n = 43 after the third. Among patients without a ≥30% reduction in pain intensity at 3 months, in STRIDE and PACE, respectively, 23.3% and 28.1% achieved a ≥30% reduction at 6 months, increasing to 33.9% and 45.7% at 12 months. Similar results were obtained when a decrease of ≥50% was used as the responder definition. Progressive improvements in pain intensity in slower responders reached levels similar to those in early responders at month 12 and were accompanied by improvements in sleep, QOL, and patient satisfaction.

**Conclusions:**

Although some patients with peripheral neuropathic pain experience rapid improvements with a single treatment of capsaicin 179 mg patch, some may require two or three treatments before an initial response is observed. Similar benefits for pain, sleep, and QOL can be achieved in early and late responders.

## Introduction

Neuropathic pain (NeP) is a common neurological disorder with an estimated worldwide prevalence of 7–10% [1]. Peripheral NeP can have significant negative consequences for patient functioning and quality of life (QOL) [2], and individuals with peripheral NeP frequently experience impaired cognition, sleep disturbances, anxiety, and depression [3, 4]. The pathophysiology of peripheral NeP is not fully understood but is thought to involve predominantly damage to sensory nerve fibers, such as small unmyelinated C fibers and myelinated A fibers (Aβ and Aδ) [4].

Capsaicin is a potent and highly selective agonist of the transient receptor potential vanilloid 1 (TRPV1), an ion channel–receptor complex expressed on skin nociceptive nerve fibers. Topical administration causes prolonged depolarization of sensory afferents, leading to defunctionalization of hyperactive nociceptors in the skin and hence to pain relief [5]. This defunctionalization is temporary, and after a period of months (depending on the concentration) following capsaicin application, sensory fiber densities are restored [6, 7].

The capsaicin 179 mg (8% w/w) cutaneous patch (Qutenza^®^ [Grünenthal GmbH, Aachen, Germany]) allows for deposition of high concentrations of capsaicin directly into the skin [5, 8]. Phase 3 trials have shown that the capsaicin 179 mg cutaneous patch provides rapid and prolonged reductions in pain across a wide variety of peripheral NeP conditions, including painful diabetic peripheral neuropathy (PDPN), post-herpetic neuralgia (PHN), and HIV-associated neuralgia (HIV-AN) [9, 10].

Data from randomized controlled trials [11–15] and real-world studies [16–18] have suggested that repeated application of the capsaicin 179 mg cutaneous patch is associated with sustained efficacy or even progressive improvements. This raises an important clinical practice question around whether continued/progressive response is limited to those who respond after the first cycle of treatment. If progressive improvements are possible even in patients who do not initially respond, a second question is raised about how many patch applications should routinely be given before judgments are made on whether to continue therapy. This is highly relevant in ensuring that as many patients as possible can benefit from this topical treatment without any central side effects. However, to the best of our knowledge, these questions have not been formally assessed.

Two phase 3/4 long-term safety trials evaluated the safety and efficacy of repeated application of the capsaicin 179 mg cutaneous patch in non-PDPN populations (STRIDE) [13] and in patients with PDPN (PACE) [14, 15]. The trials included the most prevalent peripheral NeP conditions: peripheral NeP after herpes zoster, HIV, or nerve injury (STRIDE), and PDPN (PACE). Taken together, the data collected in these trials cover treatment of mononeuropathies (e.g., PHN and post-traumatic/post-surgical nerve injury [PNI]) and polyneuropathies (e.g., HIV-AN and PDPN). The data also cover peripheral NeP with a range of etiologies: PDPN is the prototype of a peripheral NeP caused by a continuous insult, whereas PNI is the prototype of a peripheral NeP caused by a noncontinuous nerve lesion.

**Figure 1.**
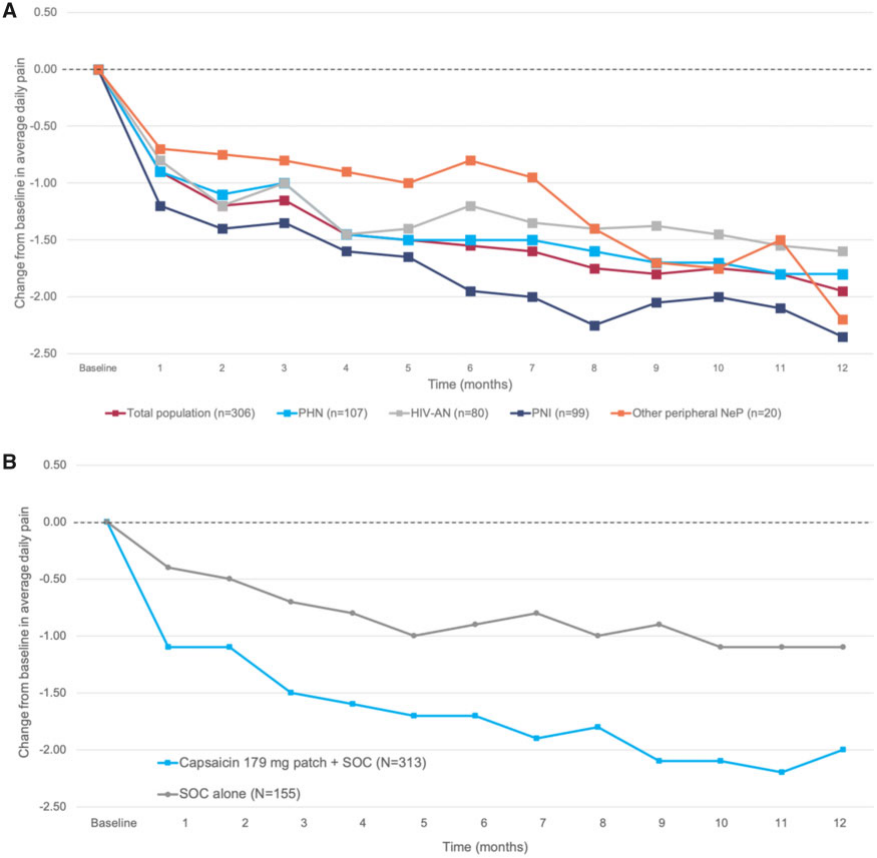
(A) Reduction in pain intensity in STRIDE. Arrows in part A show the mean day of successive capsaicin treatments and the number of patients receiving treatment. **(B)** Reduction in pain intensity in PACE.

In both trials, repeat treatment was well tolerated, with little deterioration in sensory function [13, 14]. Furthermore, average pain intensity continued to fall throughout follow-up (Figure 1), which suggests a potentially progressive response to long-term therapy. In STRIDE, this decline over time was evident in both the overall population and in diagnostic subgroups (PHN, HIV-AN, others). STRIDE and PACE also demonstrated improvements in pain scores and Patient Global Impression of Change (PGIC) [13, 15]. Furthermore, in the PACE trial in PDPN, the capsaicin 179 mg cutaneous patch was associated with improvements in QOL (measured with the EuroQol 5-dimension [EQ-5D] tool) compared with controls [15].

The assessment of the evolution of responder rates (i.e., the proportion of patients with a ≥30% reduction from baseline in average daily pain [Numeric Pain Rating Scale score]) is important to understand the contribution of each application of the capsaicin 179 mg cutaneous patch to the overall result. These results have been reported only partially for PACE and not yet for STRIDE. Given the high granularity of the data collected in these two clinical trials, it is feasible to assess the decrease in pain scores and thus responder rates after successive treatments with the capsaicin 179 mg cutaneous patch. The objectives of the present post hoc analysis of data from STRIDE and PACE were to investigate 1) whether there is any evidence of efficacy of the capsaicin 179 mg cutaneous patch in patients who did not achieve a pain response after the first application and 2) how many separate applications may be reasonable before reaching a conclusion on whether or not to continue treatment.

## Methods

### Trial Design and Procedures

This was a post hoc, exploratory analysis of data from two prospective, multinational, open-label trials of repeat treatment with the capsaicin 179 mg cutaneous patch over 52 weeks. STRIDE (NCT01252160) was a phase 4, single-arm, observational, safety trial in patients with nondiabetic peripheral NeP; all study subjects were treated with the capsaicin 179 mg cutaneous patch with repeated applications at 9- to 12-week intervals [13]. PACE (NCT01478607) was a phase 3, randomized, controlled, safety trial in patients with PDPN; participants were randomized 1:1:1 to treatment of the feet with the capsaicin 179 mg cutaneous patch (30- or 60-minute applications) combined with standard of care (SOC) or to SOC alone, and repeated applications were given at ≥8-week intervals [14, 15].

In both trials, a maximum of four patches (equivalent to an area of up to 1,120 cm^2^) were applied at any one visit. In STRIDE, applications to the feet lasted for 30 minutes, and applications to other body locations lasted for 60 minutes [13]; in PACE, all applications were to the feet and lasted for 30 or 60 minutes according to randomization group [14]. All patients were pretreated with a topical local anesthetic before patch application (although this is no longer required in the European Union [8]).

Retreatment with the capsaicin 179 mg cutaneous patch was undertaken according to investigator discretion and patient feedback (after the minimum time had elapsed since the previous treatment; i.e., STRIDE 9–12 weeks, PACE ≥8 weeks).

Both trials were approved by relevant institutional review boards and independent ethics committees, as per local requirements, and were conducted in accordance with the Declaration of Helsinki and other applicable guidelines. Written informed consent was obtained from all patients before initiation of study-related procedures.

### Participants

Full details of the inclusion and exclusion criteria for STRIDE and PACE are available elsewhere [13, 14]. Briefly, in STRIDE, eligible patients were 18–90 years of age and had a diagnosis of PHN (pain persisting since shingles vesicle crusting), PNI, or HIV-AN (confirmed with the Brief Peripheral Neuropathy Screen), all with a minimum duration of 3 months, or another adequately characterized peripheral NeP. Included patients had an average daily pain score of ≥4 (assessed with question 5 of the Brief Pain Inventory [BPI], which asks respondents to rate their average pain level over the past 24 hours on a scale of 0 [no pain] to 10 [pain as bad as can be imagined]) [13]. In PACE, all patients were ≥18 years of age, had a diagnosis of PDPN (confirmed by a score of ≥3 on the Michigan Neuropathy Screening Instrument) due to type 1 or type 2 diabetes mellitus for ≥1 year before the screening visit, and had an average daily pain score of ≥4 (assessed with question 5 of the BPI–Diabetic Neuropathy version) [14].

For the present analysis, patients were divided according to how many applications of the capsaicin 179 mg cutaneous patch were needed before they achieved a response (≥30% reduction from baseline in pain intensity, assessed with the BPI). Specifically, this assessed responders after one vs two vs three applications, and others (i.e., those with no response to a first or second application who were not offered subsequent applications, as well as those with no response to a third application). Patients who responded after a first, second, or third treatment could also receive further applications (e.g., a patient who responded after the third application may have continued and received a further two applications and therefore would have received a total of five applications).

### Assessments

The present analysis focused on the following outcomes in each group: change from baseline in average pain intensity, derived from responses to question 5 of the BPI (which asks respondents to “circle the one number that best describes your pain on the average” on a scale of 0 [no pain] to 10 [pain as bad as you can imagine]); change from baseline in mean “interference with sleep” score, derived from responses to question 9 F of the BPI (which asks respondents to rate how much their pain interfered with sleep in the prior 24 hours on a scale of 0 [did not interfere] to 10 [completely interfered]); QOL according to EQ-5D, assessed on a visual analog scale; and patient-rated Self-Assessment of Treatment (SAT), assessed at study end on a five-point Likert-type scale ranging from –2 (a strong negative response) to +2 (a strong positive response). Before each application of the capsaicin 179 mg patch, patients filled out a PGIC question in which they rated their perceived change in their condition, assessed on a scale of 1 (very much improved) to 7 (very much worse). The PGIC measures taken before an application reflected the patient’s experience of the previous application. Reasons for study discontinuation and use of concomitant pain medications (gabapentin/pregabalin or opioids) were also assessed in each responder group.

### Statistical Methods

In this post hoc analysis, descriptive statistics are provided as appropriate, including mean and standard deviation for continuous variables and frequency and percentage for categorical variables. Missing data were not imputed.

## Results

### Baseline Characteristics and Treatments

In STRIDE, 306 patients received treatment with the capsaicin 179 mg cutaneous patch (PHN, n = 107; PNI, n = 99; HIV-AN, n = 80; other peripheral NeP conditions, n = 20). PACE included 468 patients with PDPN randomized to the capsaicin 179 mg cutaneous patch (30 minutes) plus SOC (n = 156), to the capsaicin 179 mg cutaneous patch (60 minutes) plus SOC (n = 157), or to SOC alone (n = 155). In the present analysis, data from PACE were evaluated only for the two groups that received the capsaicin 179 mg cutaneous patch (30 or 60 minutes) plus SOC (n = 313).

Full baseline characteristics are described elsewhere [13, 14]. In both trials, there were no apparent differences between responder groups in sex, age, duration of peripheral NeP, and mean pain at baseline (Table 1).

**Table 1.** Baseline characteristics by responder category in STRIDE and PACE

Characteristic	Total	Responders After First Application	Responders After Second Application	Responders After Third Application	Others*
STRIDE	N = 306	n = 60	n = 33	n = 11	n = 202
Sex, female, n (%)	132 (43.1)	28 (46.7)	19 (57.6)	7 (63.4)	78 (38.6)
Age, years, mean ± SD	57.9 ± 15.0	58.7 ± 15.8	60.5 ± 15.4	55.0 ± 13.1	57.3 ± 14.9
Peripheral NeP duration, years, mean ± SD	5.1 ± 5.5	5.2 ± 5.5	4.4 ± 4.3	5.1 ± 5.9	5.2 ± 5.6
Baseline pain^†^, mean ± SD	6.6 ± 1.4	6.3 ± 1.3	6.6 ± 1.1	6.1 ± 1.3	6.8 ± 1.5
PACE	N = 313	n = 96	n = 68	n = 43	n = 106
Sex, female, n (%)	160 (51.1)	54 (56.3)	35 (51.5)	20 (46.5)	51 (48.1)
Age, years, mean ± SD	61.0 ± 10.6	58.9 ± 10.6	61.3 ± 11.8	61.6 ± 9.0	62.3 ± 10.2
Peripheral NeP duration, years, mean ± SD	4.3 ± 3.8	4.5 ± 4.9	4.2 ± 3.5	3.7 ± 2.7	4.4 ± 3.1
Baseline pain^†^, mean ± SD	5.6 ± 1.3	5.6 ± 1.2	5.6 ± 1.4	5.6 ± 1.1	5.6 ± 1.5

SD=standard deviation.

* Includes nonresponders to first or second application who were not offered subsequent applications, as well as nonresponders to third application.

† Assessed with question 5 of the BPI.

### Pain Intensity

**Figure 2.**
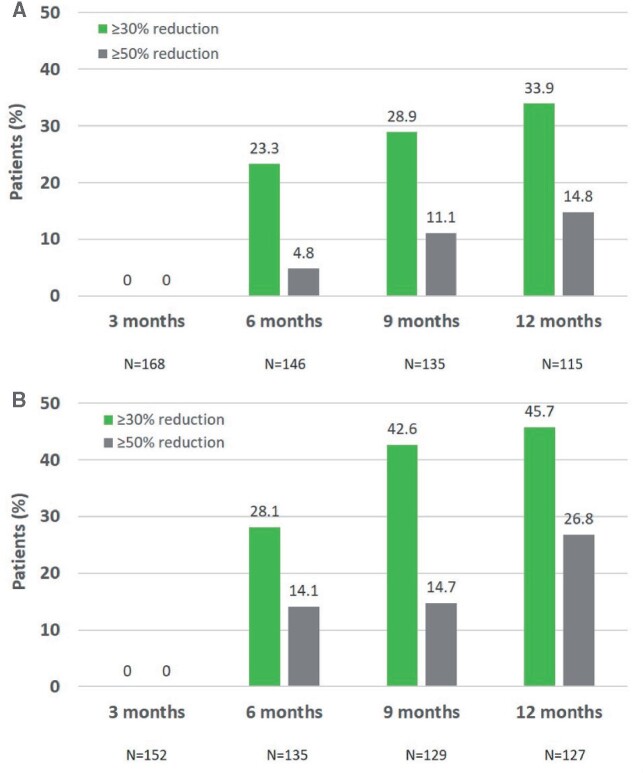
(A) Rates of responders among initial nonresponders in STRIDE. **(B)** Rates of responders among initial nonresponders in PACE. The figure shows the proportion of patients who went on to achieve a ≥30% or ≥50% reduction in pain intensity (assessed with question 5 of the BPI) after having failed to achieve a ≥30% response at 3 months.

Among patients who did not have a 30% reduction from baseline in pain intensity 3 months after the first treatment, approximately one quarter were responders at 6 months (STRIDE, 23.3%; PACE, 28.1%), and more than a third had a response at 12 months (STRIDE, 33.9%; PACE, 45.7%) (Figure 2). Similarly, substantial numbers of patients who did not have a ≥30% reduction from baseline in pain intensity at 3 months went on to achieve a ≥50% response at later time points.

**Figure 3.**
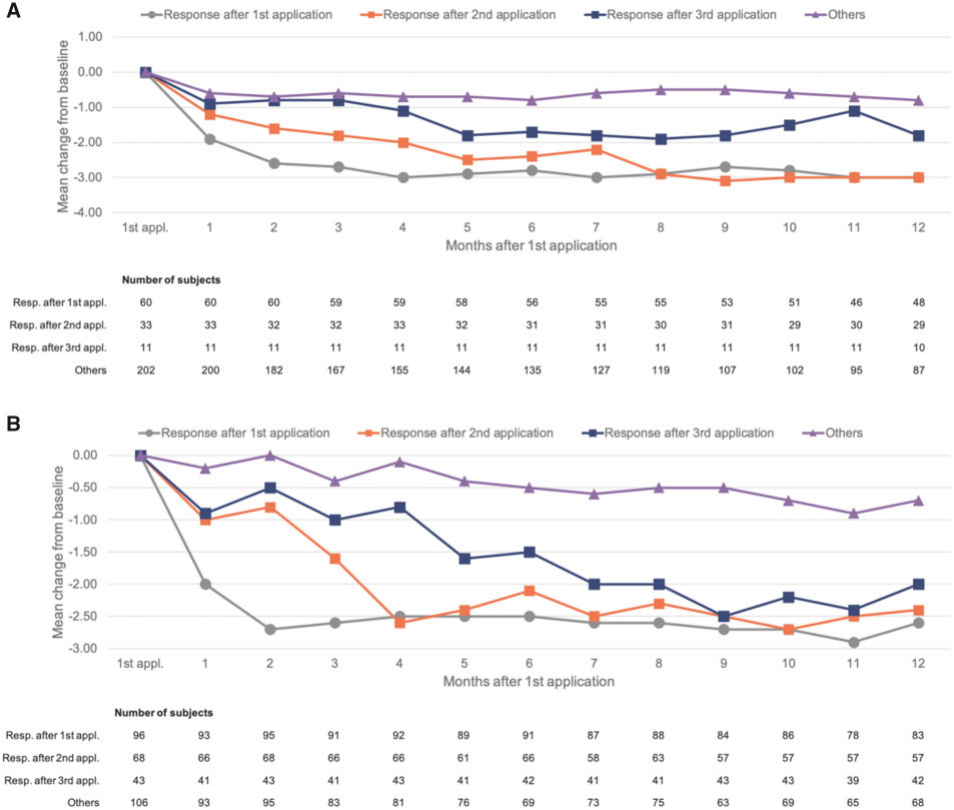
(A) Change from baseline in pain intensity by responder category in STRIDE. **(B)** Change from baseline in pain intensity by responder category in PACE. Pain intensity scores are derived from question 5 of the BPI, which asks respondents to rate their average pain over the prior 24 hours on a scale of 0 (no pain) to 10 (pain as bad as can be imagined). “Other” includes nonresponders to first or second application who were not offered subsequent applications, as well as nonresponders to third application.

An analysis of changes in pain intensity by responder category demonstrated the expected rapid responses (within 1–2 months) in patients who responded after the first application of the capsaicin 179 mg cutaneous patch (Figure 3). These responses were then sustained throughout follow-up. In both trials, those who achieved a response only after the second application showed a slower decline in pain intensity but ultimately achieved a response similar to that of the initial responders. In PACE, those who achieved a response only after the third application also ultimately achieved similarly deep responses after around 7–9 months (Figure 3B); there were few such patients in STRIDE (n = 11), but the data were suggestive of progressive (albeit partial) responses (Figure 3A).

### Other Efficacy End Points

**Figure 4.**
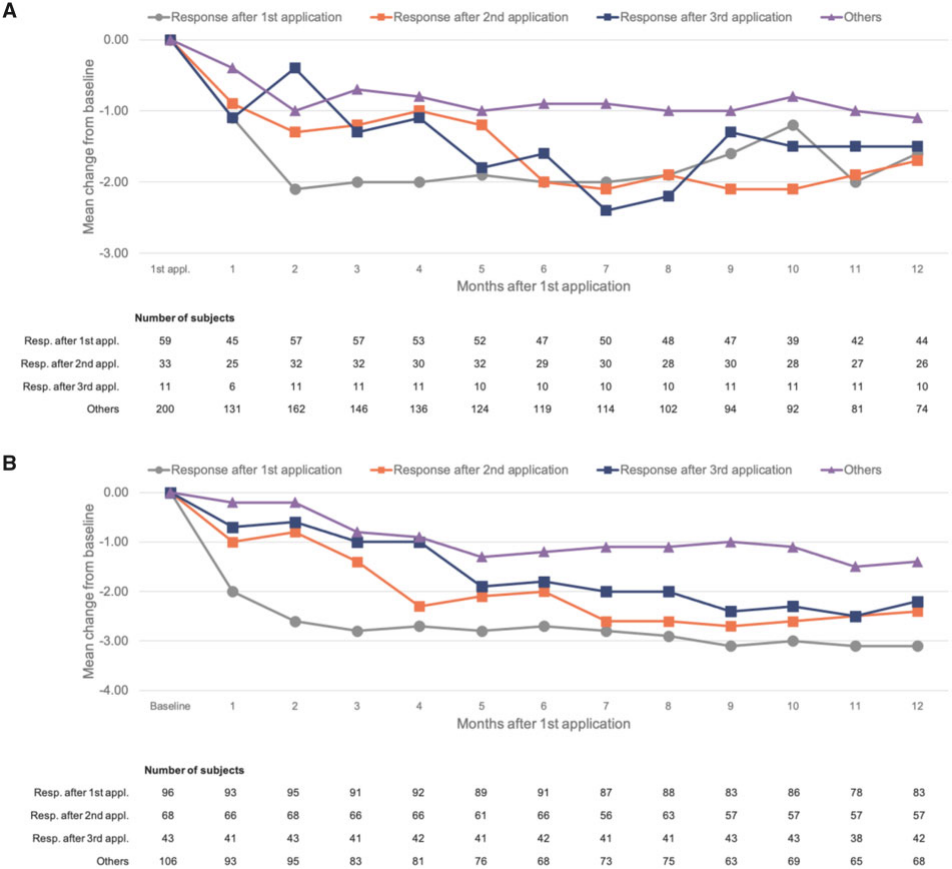
(A) Change from baseline in “interference with sleep” score by responder category in STRIDE. **(B)** Change from baseline in “interference with sleep” score by responder category in PACE. Scores are derived from question 9 F of the BPI, which asks respondents to rate how much pain interfered with their sleep in the prior 24 hours on a scale of 0 (did not interfere) to 10 (completely interfered). “Other” includes nonresponders to first or second application who were not offered subsequent applications, as well as nonresponders to third application.

Reductions in sleep interference from baseline by responder category showed a pattern similar to that of the declines in pain intensity (Figure 4). There were rapid reductions in sleep interference (within 1–2 months) in patients with a pain response after the first application of the capsaicin 179 mg cutaneous patch, and these responses were sustained throughout follow-up. Declines in sleep interference were slower in patients who achieved a pain response only after the second application (∼6–7 months), but were similarly deep to those with a pain response after the first application. Reductions in sleep interference were slower again in those achieving a pain response only after three applications—but appeared ultimately to be almost as deep as those among rapid responders.

**Figure 5.**
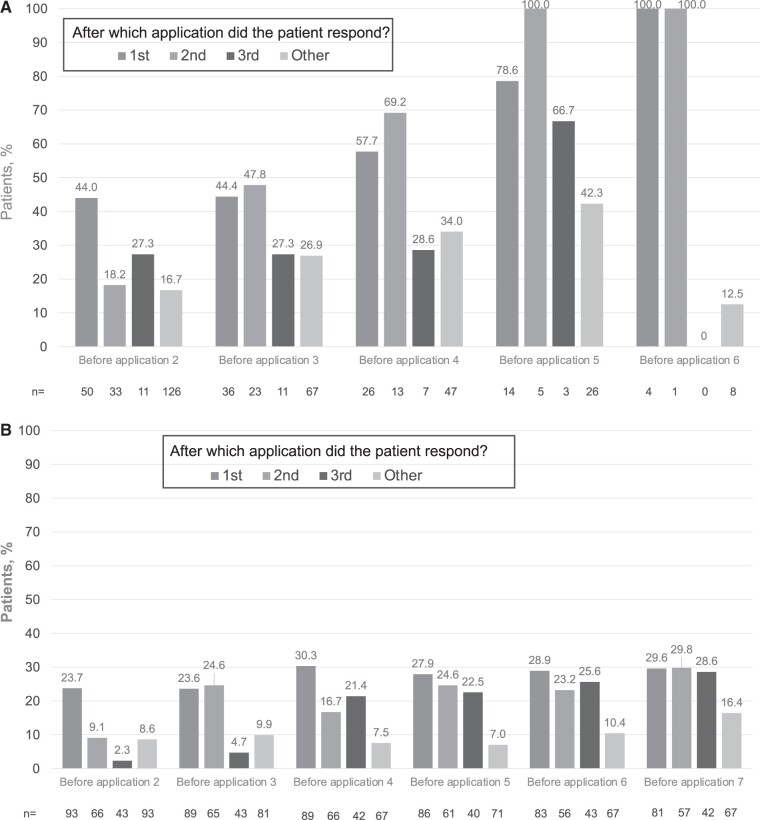
(A) Patients rating change from baseline on PGIC as “very much improved” or “much improved” in STRIDE. **(B)** Patients rating change from baseline on PGIC as “very much improved” or “much improved” in PACE. “Other” includes nonresponders to first or second application who were not offered subsequent applications, as well as nonresponders to third application; PGIC measures taken before an application reflect the patient’s experience of the previous application.

The proportions of patients who reported being “very much improved” or “improved” on PGIC also paralleled the improvements in pain (Figure 5). Figure 5 shows the proportions of patients who reported being “very much improved” or “improved” on PGIC at each time point at which this question was administered, i.e., before each application, and this is shown according to the response group (response to first, second, third, or other). For example, in PACE, 23.7% of pain responders after one application had improved on PGIC at month 2, compared with only 9.1% and 2.3% of responders after two or three applications, respectively. Responders after the second and third applications showed similar levels of PGIC improvements only at months 4 and 6, respectively. By the end of the PACE trial, rates of improvement on PGIC were similar across all three responder groups and substantially higher than among other patients (nonresponders to first or second application who were not offered subsequent applications, and nonresponders to third application) (Figure 5).

Improvements in patient QOL assessed with the EQ-5D followed a similar trend, albeit less clear than with PGIC.

**Figure 6.**
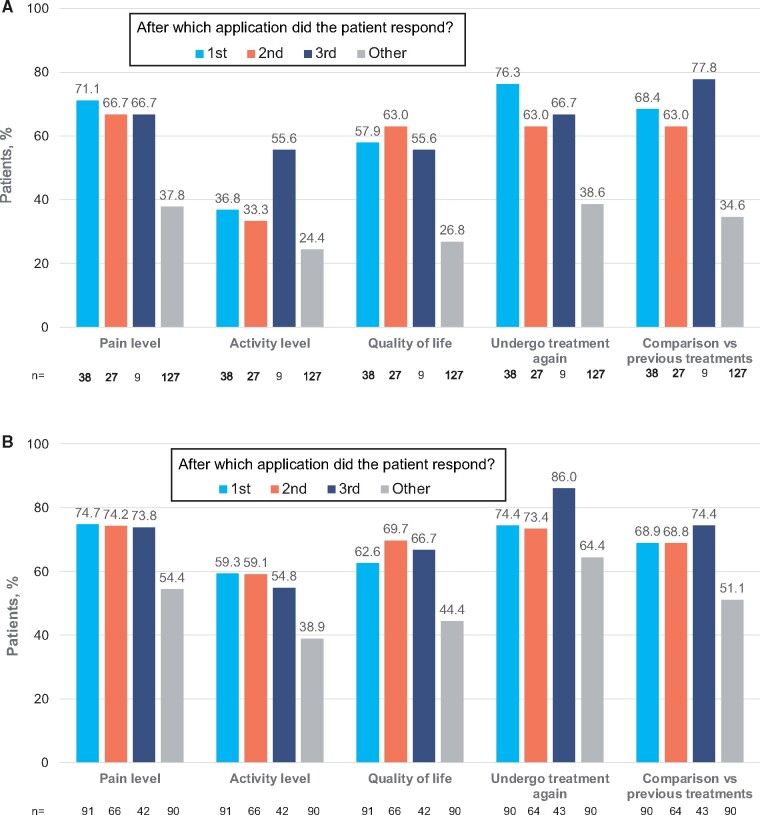
(A) Percentage of patients reporting positive responses to each SAT domain in STRIDE. **(B)** Percentage of patients reporting positive responses to each SAT domain in PACE. Positive response defined as patients responding with a +1 or +2 for each domain of SAT on a five-point Likert-type scale ranging from –2 (a strong negative response) to +2 (a strong positive response). “Other” includes nonresponders to first or second application who were not offered subsequent applications, as well as nonresponders to third application.

Rates of patient satisfaction (assessed at the end of each trial) were higher for all five domains of SAT among responders than among other patients (nonresponders to first or second application who were not offered subsequent applications, and nonresponders to third application), irrespective of whether patients needed one, two, or three applications of the capsaicin 179 mg cutaneous patch to achieve a response (Figure 6). This suggests that late responders “caught up” with those who experienced a rapid response.

### Treatment Discontinuations and Concomitant Pain Medications

As would be expected, treatment discontinuations among nonresponders in both trials resulted primarily from lack of efficacy or withdrawal of consent (Table 2). In both trials, discontinuation rates were low among responders, and there were no obvious differences in discontinuation profiles among those responding after one, two, or three applications of the capsaicin 179 mg cutaneous patch.

**Table 2.** Reasons for discontinuation by responder category in STRIDE and PACE

Reason	Total	Responders* After First Application	Responders* After Second Application	Responders* After Third Application	Others^†^
STRIDE	N = 306	n = 60	n = 33	n = 11	n = 202
Adverse event	11 (3.6)	2 (3.3)	0	1 (9.1)	8 (4.0)
Lack of efficacy	54 (17.6)	4 (6.7)	2 (6.1)	1 (9.1)	47 (23.3)
Protocol violation	6 (2.0)	1 (1.7)	0	0	5 (2.5)
Lost to follow-up	17 (5.6)	1 (1.7)	2 (6.1)	1 (9.1)	13 (6.4)
Withdrawal of consent	33 (10.8)	3 (5.0)	1 (3.0)	0	29 (14.4)
Other	9 (2.9)	0	0	0	9 (4.5)
PACE	N = 313	n = 96	n = 68	n = 43	n = 106
Adverse event	15 (4.8)	3 (3.1)	3 (4.4)	0	9 (8.5)
Lack of efficacy	4 (1.3)	2 (2.1)	0	0	2 (1.9)
Protocol violation	4 (1.3)	0	0	0	4 (3.8)
Lost to follow-up	3 (1.0)	0	1 (1.5)	0	2 (1.9)
Withdrawal of consent	25 (8.0)	4 (4.2)	2 (2.9)	0	19 (17.9)
Other	2 (0.6)	2 (2.1)	0	0	0

All data are n (%).

* Response was defined as ≥30% reduction from baseline in pain intensity, assessed with the BPI.

† Includes nonresponders to first or second application who were not offered subsequent applications, as well as nonresponders to third application.

**Figure 7.**
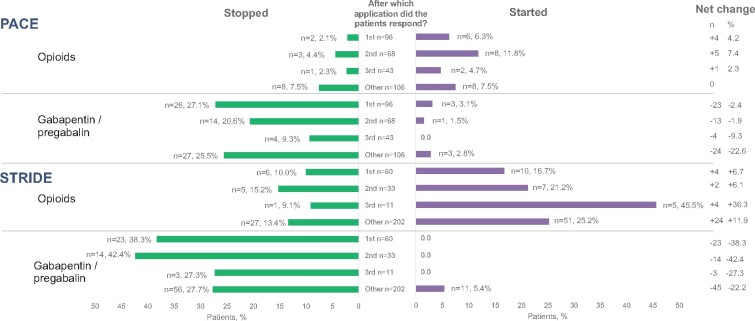
Percentage of patients who stopped or started gabapentin/pregabalin or opioids according to responder group in PACE and STRIDE. “Stopped” indicates using at baseline but not using after baseline; “Started” indicates not using at baseline but using after baseline; “Other” includes nonresponders to first or second application who were not offered subsequent applications, as well as nonresponders to third application.

Differential use of concomitant pain medication is a potential confounder of the analysis of responses. Across trials and in all three subgroups, pregabalin or gabapentin were more often discontinued than initiated. Opioids were initiated in a slightly higher proportion than discontinued in those responding after the first and second treatment across trials (Figure 7), and hence there are no data to suggest that greater use of other pain medications could explain apparent responses in those who responded only after the second or third application of the capsaicin 179 mg cutaneous patch.

### Treatment Intervals

Throughout STRIDE, the mean interval between retreatments was 105.4 days. It appeared to be longer in patients who responded after one application (116.7 days) than in those who responded after two (103.1 days) or three applications (94.5 days). The mean interval between retreatments in nonresponders was 103.5 days.

In PACE, the mean interval between retreatments was 66.6 days, and it was similar in patients who responded after one application (65.9 days), two applications (65.1 days), or three applications (63.8 days) and also in nonresponders (69.9 days).

## Discussion

It has been previously suggested that effectiveness further increases with repeated applications of a capsaicin 179 mg cutaneous patch [15]; however, no systematic assessment of additional benefit from repeated treatments with the capsaicin 179 mg cutaneous patch has been reported to date. This post hoc exploratory analysis of data from two phase 3/4 trials of the capsaicin 179 mg cutaneous patch provides the first evidence in support of additional benefit for patients with repeated applications, irrespective of initial response. Although some individuals experienced rapid and sustained responses after initial treatment, others demonstrated a more progressive pattern of response after a second or third application, with incremental improvements in pain and other efficacy end points. This effect did not appear to result from confounding effects of baseline characteristics, treatment discontinuations, or concomitant pain medications, given that these profiles did not differ substantially among response groups. Thus, the present work provides a rationale for trying a second and possibly a third application of the capsaicin 179 mg cutaneous patch in patients who do not respond to the first application, before deciding whether or not to continue treatment.

Previous studies investigating possible predictors of response to the capsaicin 179 mg cutaneous patch were based on randomized controlled clinical trials focusing on a single application, not taking into account that some patients may not respond to a single treatment but become responders with subsequent applications [19, 20]. The predictors found were baseline pain scores, variability of pain before treatment, pain response to lidocaine pretreatment, and time with preexisting pain, but the predictors may vary according to the etiology of the peripheral NeP [20].

However, recently it was noted by clinicians that, in clinical practice, patients can benefit from repeated applications irrespective of response to a first application. This concept of progressive improvement is not inconsistent with the pattern of response observed with other treatments associated with chemodenervation. For example, in The Chronic migraine OnabotulinuMtoxinA Prolonged Efficacy open-Label (COMPEL) Study trial, repeated use of onabotulinumtoxin A in chronic migraine was associated with continued gradual improvements in response over the course of nine treatment cycles across 2 years [21]. Furthermore, although not associated with chemodenervation, use of monoclonal antibodies against anti-calcitonin gene–related peptide (CGRP) or its ligand in the prevention of migraine is associated with continued improvement over time [22–24].

A recent study of the capsaicin 179 mg cutaneous patch in patients with chemotherapy-induced peripheral neuropathy provides a potential mechanistic explanation for the progressive response [25]. In that study, skin biopsies taken before and 3 months after patch application demonstrated significant increases toward normalization of intra-epidermal and sub-epidermal nerve fibers (for the structural marker PGP9.5, heat receptor TRPV1, and regenerating nerve marker GAP43). In addition, epidermal levels of nerve growth factor, neurotrophin-3, and Langerhans cells were also normalized [25]. The authors therefore proposed that the capsaicin 179 mg cutaneous patch may have disease-modifying as well as pain-relieving effects, hypothesizing that patch application leads to “pruning” of abnormal nerve fibers—thus inducing nerve regeneration and restoration of more normal nerve fiber phenotypes [25]. This gradual restoration of function is a possible explanation for the progressive pattern of response seen the present study and the reason why, in some patients, a response is observable only after a second or third application.

Nociceptor sensitization over time [26] may also play a role: If the TRPV1 receptors become sensitized as a consequence of tissue insult and inflammation, previously ineffective stimuli may become effective. There may also be a gradual effect of peripheral therapy on central sensitization, leading to decreased perception of pain over time. Finally, gradual improvements in pain after application of the capsaicin 179 mg patch could lead to increased patient activity and thus to further improvements in pain. The specific insult leading to pain varies according to the type of NeP, and underlying mechanisms may therefore differ. Nonetheless, further exploration is warranted with regard to the impact of potential capsaicin-mediated disease-modification pathways in other NeP conditions.

The present analysis also found improvements in sleep interference with the capsaicin 179 mg cutaneous patch that followed a time course similar to that of the reductions in pain intensity. This suggests that pain reductions may have been the main reason for sleep improvements. However, TRPV1 blockade has been associated with altered sleep modulation in animal models [27], and hence pain-independent mechanisms cannot be ruled out.

In the original publication of STRIDE, it was noted that the area affected by NeP decreased with repeat applications [13]. This interesting and clinically relevant finding could not be further explored because these data were not collected during PACE.

We should acknowledge the key limitations of the present work. Most importantly, it was a post hoc exploratory analysis, and prospective assessments will be required to confirm the value of repeat application of the capsaicin 179 mg cutaneous patch in patients who do not respond to the first cycle of treatment. The analysis was “as treated,” and only patients who received at least one application were included. Missing data from patients who withdrew from the studies could not be accounted for, and this is a study limitation. In this analysis, there were no comparative data, and hence there was no way to determine which improvements were due to therapy and which were attributable to other factors, such as the Hawthorne effect (changes in behavior among patients included in a trial) [28], natural resolution of pain, and background care.

In a subgroup analysis of an open-label trial designed to study the tolerability of multiple treatments of the capsaicin 179 mg cutaneous patch over 52 weeks, patients were divided in subgroups according to the duration of NeP since diagnosis [16]. That analysis showed that earlier treatment with the capsaicin 179 mg cutaneous patch resulted in better treatment outcomes. Our study did not include a formal analysis of a differential response according to duration of NeP.

It is logical that patients who did not respond early might have started other pain medications, and this is a potential confounding factor. However, in STRIDE, there were no patients among the responder groups who started using gabapentin/pregabalin after baseline; in PACE, there were only four such patients. Use of these pain medications thus is not likely to have confounded the results. With regard to opioids, some patients started opioids, but others stopped taking them, which suggests that, for certain patients, the capsaicin 179 mg cutaneous patch provided an opioid-sparing effect. If opioid initiation were a significant confounder, we would expect the net change in opioid use to be greater in the groups of patients who responded late (after the second or third application) than in the patients who responded early. In STRIDE, the net change in opioid use in patients who responded to the first, second, and third application was +4 (+6.7%), +2 (+6.1%), and +4 (36.3%) patients, respectively. In PACE, the net change in opioid use in patients who responded to the first, second, and third application was +4 (+4.2%), +5 (+7.4%), and +1 (2.3%) patients, respectively. Given the apparent similarity of the net change in opioid use among the responder groups [29, 30], we conclude that initiation of opioids was not a major confounding factor in the present analysis.

It is not clear why discontinuations were more common in STRIDE than in PACE. However, it is possible that there was less incentive to remain in STRIDE because patients were able to access the treatment outside the trial, as the NeP types included in STRIDE were within the approved indication at the time.

## Conclusions

Some patients experience rapid and long-lasting improvements in pain after a single local treatment of peripheral NeP with the capsaicin 179 mg cutaneous patch, whereas others may follow a more progressive or incremental course of benefit in terms of pain, sleep, QOL, and patient satisfaction. In the latter individuals, two or even three treatments ≥8 weeks apart are needed before a response is achieved. Slower responders appear to “catch up” with rapid responders over time. Repeat treatment may therefore be important to maximize efficacy in those who do not initially respond.

The capsaicin 179 mg cutaneous patch was also generally well tolerated and was associated with low levels of discontinuations due to adverse events in all subgroups studied.

The potential mechanisms underlying progressive responses to the capsaicin 179 mg cutaneous patch are not fully understood, but they may relate to disease modification through “pruning” of abnormal nerve fibers, followed by regeneration of more normal fibers [25], and this effect may become more pronounced with each successive treatment. Further studies are warranted to analyze these mechanisms in different peripheral NeP conditions.

## Notes

Funding sources: The STRIDE and PACE trials were sponsored by Astellas Pharma Inc. Grünenthal GmbH sponsored the present analysis and funded medical writing support for this article.

Conflicts of interest: Rainer Freynhagen has received consultancy and speaker fees within the past 2 years from AOP Orphan, Eli Lilly and Company, Hikma Pharmaceuticals, Grünenthal GmbH, Merck Consumer Health, Mitsubishi Tanabe Pharma, Pfizer, Pfizer Upjohn, P&G Health Germany GmbH, Roche Pharma, and SCILEX Pharmaceuticals, outside of the submitted work. Charles Argoff has served as a consultant to Salix, AstraZeneca, Shionogi, BDSI, Vertex, Lilly, Teva, Novartis, US WorldMeds, Pfizer, and Flowonix, and as a speaker to US WorldMeds, DSI, Amgen, Novartis, Scilex, Lilly, and Teva. Marielle Eerdekens and Sylvia Engelen are employees of Grünenthal GmbH. Serge Perrot has received fees for consultancy from Pfizer, Lilly, MSD, UPSA, Grünenthal, and Sanofi and research grants from Grünenthal.
